# Improving Usability of the Interrupting Prolonged Sitting With Activity Virtual Teacher Training Modules: Case Study

**DOI:** 10.2196/83427

**Published:** 2026-04-21

**Authors:** Rebecca E Hasson, Tiffany Tam, Lucy Myers, Anna Schwartz, Alissa Li

**Affiliations:** 1 School of Kinesiology, University of Michigan, 830 North University Avenue, 4220 SKB, Ann Arbor, MI, 48109, United States, 1 7347638671; 2 School of Information, University of Michigan, Ann Arbor, MI, United States

**Keywords:** user experience, website, implementation science, human-centered design, HCD, web-based, interview, heuristic, competitive analysis, classroom physical activity, teachers

## Abstract

**Background:**

High-quality professional development can help teachers build the skills and confidence needed to implement evidence-based practices, such as classroom physical activity breaks. While in-person training is often preferred, virtual asynchronous training offers a flexible alternative for teachers. However, its effectiveness may be limited by design and usability challenges.

**Objective:**

The aim of this study was to conduct a usability assessment of the Interrupting Prolonged Sitting with Activity (InPACT) virtual teacher training modules, using a human-centered design (HCD) approach to align the training with end-user preferences and needs.

**Methods:**

The InPACT professional development program includes nine modules delivered through an online platform (Qualtrics XM). A usability assessment was conducted using (1) structured online surveys with elementary school teachers who had completed the modules, (2) a heuristic evaluation based on the Jakob Nielsen 10 usability heuristics, and (3) a competitive analysis of three learning management systems (Moodle, Teachable, and Thinkific) to identify platform strengths, limitations, and insights. Findings and recommendations were compiled to inform module improvements.

**Results:**

Eleven teachers completed the survey. They reported that the modules were easy to use, the content was informative and relevant, and they valued the interactive and practical components. Areas for improvement included enhancing content engagement and increasing technical flexibility. The heuristic evaluation identified 14 usability issues across nine of the Nielsen heuristics (eg, navigation difficulties and text-heavy pages). The competitive analysis highlighted features that enhance user experience, such as progress tracking, estimated completion times, interactive elements, and feedback on quiz answers.

**Conclusions:**

Usability assessments grounded in an HCD approach can enhance virtual training for educators, improving the uptake and implementation of evidence-based practices, such as classroom physical activity breaks. Five overarching recommendations emerged as follows: (1) removing video time constraints, (2) implementing accurate progress bars, (3) incorporating active learning or retention activities, (4) creating consistent and actionable end pages, and (5) ensuring consistency in titles and references to physical materials. Building on preliminary positive data from revised modules, future research should evaluate the impact of HCD revisions on teacher self-efficacy, training completion, and fidelity of program implementation.

## Introduction

Physical activity plays a vital role in promoting child and adolescent health by improving cognition, bone health, fitness, and overall health, while also reducing the risk of anxiety and depression [1]. However, most children and adolescents in the United States do not meet the Physical Activity Guidelines for Americans, which recommend at least 60 minutes of moderate-to-vigorous physical activity daily to attain the greatest health benefits [1]. National surveillance data show that only 42% of children aged 6 to 11 years meet this recommendation [2], with participation declining sharply during adolescence [3] and rates being even lower for youth residing in resource-limited communities [4]. This shortfall increases the risk of obesity and related chronic conditions [5,6], highlighting the need to improve youth physical activity engagement.

Schools offer a critical and equitable setting to help close the physical activity gap among children and adolescents [7,8]. Among school-based strategies, classroom-based physical activity interventions have emerged as effective and feasible approaches for increasing students’ daily activity levels, particularly in resource-limited settings [9,10]. These interventions provide structured opportunities for all children to engage in regular exercise by integrating short bouts of movement into the school day. Compared to physical education and after-school sports programs, they are less resource-intensive and easier to implement, making them well-suited for schools with limited staff, space, or funding [11,12].

Recognized as an evidence-based practice by the Community Preventive Services Task Force in 2021, classroom physical activity interventions typically involve teachers incorporating 1 to 5 activity breaks per day, each lasting 4 to 10 minutes [13]. Core implementation components include dedicated time, adequate classroom space, and access to age-appropriate videos and exercise resources. A systematic review and meta-analysis by the Task Force, which included 22 studies (11 using direct observation), found that physical activity breaks significantly increased students’ activity levels during school hours and improved time-on-task by a median of 26.2 minutes [10]. These findings provide strong evidence that classroom physical activity interventions can simultaneously promote physical activity and enhance classroom engagement.

Despite the well-established benefits, national data show that fewer than half of US elementary teachers incorporate these breaks regularly [14,15], underscoring the need to better understand barriers and facilitators to their implementation. While many teachers recognize these benefits—such as increased student focus, enjoyment, and a more positive classroom climate—they often experience a lack of confidence, uncertainty about how to integrate activity without disrupting instruction, and the belief that physical activity is not aligned with academic goals [16-21]. Key individual-level factors that influence implementation include teachers’ knowledge, attitudes, self-efficacy, and perceptions of social norms [22-24]. Educators who feel confident in their ability to use physical activity, value its role in learning, and perceive support from peers and administrators are more likely to use activity breaks consistently [25-27]. Strengthening these individual factors is critical to expanding the reach and sustainability of classroom physical activity.

To overcome these barriers—particularly teachers’ lack of confidence and uncertainty about how to integrate activity without disrupting learning—high-quality professional development is essential [28]. For physical activity integration to become routine, teachers need concrete, classroom-ready strategies that align with existing instructional practices [29]. The Interrupting Prolonged Sitting with Activity (InPACT) intervention addresses this need by providing a skills-based professional development program designed to support direct translation to classroom practice [30]. Through structured training, InPACT equips educators with the tools and strategies needed to integrate physical activity in ways that are feasible within daily classroom routines.

Grounded in the Guskey [28] model of teacher change, which emphasizes practice as a precursor to belief change, the InPACT approach prioritizes the adoption of new classroom behaviors before targeting shifts in attitudes or perceptions. As teachers observe improvements in student focus, classroom climate, and learning, these practice-based successes reinforce the feasibility and value of integrating movement into the school day, thereby supporting subsequent changes in beliefs and acceptability.

The educational strategies embedded within the InPACT training were intentionally selected to reflect adult learning principles and evidence-based models of effective teacher professional development [31,32]. These strategies include practical, classroom-ready examples, active skill-building, structured reflection, and explicit connections between training content and teachers’ daily instructional practices. The training was co-created with the University of Michigan School of Education’s Instructional and Program Design Coordinator, ensuring alignment with evidence-based pedagogy and responsiveness to teacher-identified needs [30].

The InPACT professional development program consists of 9 interactive training modules that can be delivered either in-person or asynchronously via an online learning platform (Qualtrics XM). In person, the modules are delivered by a trained InPACT professional using a Microsoft PowerPoint presentation, with interactive opportunities for hands-on activities, modeling of classroom activity breaks, and group discussions. In-person InPACT training sessions typically last 6 to 8 hours. The virtual format consists of asynchronous modules combining text, videos, images, and interactive exercises, with each module taking approximately 45 to 60 minutes to complete. Teachers are encouraged to complete all modules within a 4- to 6-week period to support continuity and application to classroom practice. Participants engage in guided self-reflection activities and complete premodule and postmodule assessments to track learning progress. Table 1 provides a description of each training module.

**Table 1.** Summary of the Interrupting Prolonged Sitting with Activity (InPACT) virtual teacher training modules.

Module	Title	Description
1	Evidence base for classroom physical activity	Introduces the evidence supporting classroom physical activity and its benefits for health, well-being, and academic performance.
2	Overview of InPACT	Explains how to incorporate short movement breaks (InPACT) into the school day.
3	Optimizing classroom layouts	Guides teachers in creating safe and functional spaces for movement in the classroom.
4	Classroom management during activity breaks	Focuses on maintaining structure and engagement during physical activity breaks.
5	Integrating physical activity with academic content	Helps teachers align movement with learning goals to support academic achievement.
6	Selecting exercise videos and assessing intensity	Covers selecting appropriate videos and assessing student intensity levels to meet physical activity goals.
7	Motivational techniques and gamification	Introduces strategies like gamification to enhance and sustain student engagement.
8	Inclusive adaptation of physical activity	Provides strategies to adapt activities to ensure accessibility and inclusion for all students.
9	Safety guidelines	Emphasizes safety considerations and offers best practices for injury prevention and safety planning.

After developing this content-rich program, we sought to understand teachers’ preferences for how these modules could best be delivered. Findings from a group of general education teachers (n=35, teaching experience ranging from 2 to 27 years; mean 14.8, SD 7.4) highlighted the importance of offering flexible training options for classroom physical activity implementation. While nearly two-thirds of teachers expressed a preference for in-person professional development, a substantial portion—approximately one-third—preferred a virtual, asynchronous training option. These teachers cited competing demands, stress, and logistical challenges (such as preparing substitute teacher plans) as barriers to in-person participation (Friday et al, PhD, unpublished data, 2021). Providing autonomy in training modality—allowing educators to select either virtual or in-person formats—could therefore enhance participation and reduce perceived burden, particularly if training is integrated into existing professional development sessions.

Offering virtual training may increase accessibility for teachers facing time constraints and logistical challenges. However, it also introduces a new set of considerations—particularly regarding the usability of the digital learning environment. Ensuring that these online modules are accessible, understandable, and engaging is essential to addressing the very barriers—such as stress and overload—that teachers have identified.

Teachers’ ability to benefit from online modules depends on the usability and design quality of the digital learning environment [33]. Usability is defined as “a quality attribute assessing how easy user interfaces are to use, focusing on 5 core components: learnability, efficiency, memorability, error handling, and satisfaction” [34]. Research shows that poorly designed websites can hinder engagement, leading to frustration and high dropout rates [35]. Conversely, user-friendly platforms can promote greater interaction and retention [33]. To address this, we conducted a usability assessment of the InPACT virtual teacher training modules to evaluate how accessible, understandable, and engaging they are for classroom educators. Ultimately, improving the usability of the InPACT virtual modules can empower more educators to confidently and sustainably implement physical activity breaks, regardless of the training format they choose.

## Methods

### Overview

This study used a mixed methods approach grounded in a human-centered design (HCD) framework to evaluate the usability and delivery of the InPACT virtual teacher training modules, with an emphasis on understanding the end-user needs, constraints, and contexts prior to large-scale implementation, consistent with established HCD models for digital health interventions [36]. Within the HCD lifecycle, this study focused on formative inspiration and ideation stages to inform iterative refinement of the training modules, operationalized through complementary methods, including a structured online survey of teachers, a heuristic usability evaluation, and a competitive analysis of learning management systems (LMSs). HCD served as the overarching, iterative, and participatory framework, integrating principles from user-centered design, which prioritizes usability and functionality from the end-user perspective, and user experience (UX) design, which focuses on optimizing the quality of users’ interactions with a system. Together, these principles informed design decisions related to the structure, usability, and delivery of the virtual modules. Later stages of HCD, including implementation and outcome evaluation, were beyond the scope of this study and are the focus of ongoing and future work.

### Recruitment

Elementary school teachers who had completed at least one InPACT virtual module were recruited for this study. Principals in Michigan schools currently implementing InPACT provided the contact information of teachers who participated in the training. Eligibility was determined through these principal-provided contact lists. Eligible teachers were invited via email to participate in the research, with a link to a structured online survey. Participants could opt out at any time.

### Structured Online Survey

A structured online survey was administered to evaluate teachers’ experiences with the InPACT intervention. Developed by the research team, the survey included both closed-ended and open-ended items to capture quantitative indicators of module use and qualitative feedback on UX. The first section gathered demographic information, such as the grade level taught and years of teaching experience. The second section assessed teachers’ experiences with the InPACT modules, including challenges encountered, clarity of module content, perceived strengths and weaknesses, preferred format of the program guide (ie, the written version of the modules; physical book vs digital pdf), use of supplementary links, suggested improvements, and frequency of classroom activity implementation following training.

All participants completed the same set of structured questions, designed to take approximately 15 to 20 minutes. Responses were collected anonymously and analyzed by the research team. Participants were offered the chance to enter a US $25 gift card raffle as an incentive for completing the survey. The full survey instrument, including all closed-ended and open-ended items, is provided in Multimedia Appendix 1.

### Heuristic Evaluation

A heuristic evaluation was conducted to assess the usability of the InPACT virtual teacher training modules. The evaluation team consisted of 5 evaluators with diverse academic backgrounds and varying levels of expertise in UX design. UX design is defined as “the process of designing digital or physical products with a focus on optimizing users’ overall experience, including usability, accessibility, and satisfaction” [37]. Some evaluators had completed formal coursework in UX design, while others contributed complementary perspectives, including spatial and visual design and applied analytics. All evaluators had prior hands-on experience conducting usability evaluations through Michigan Open UX, the University of Michigan’s student organization focused on applied UX research and practice.

Heuristic evaluation is a cost-effective usability inspection method in which evaluators assess an interface against established usability principles, or heuristics, to identify potential design issues [38,39]. This evaluation was guided by the Jakob Nielsen 10 usability heuristics [40], which are grounded in cognitive psychology and human-computer interaction principles. Each evaluator independently completed the virtual training modules and documented any usability issues encountered. Identified issues were mapped to the relevant heuristic and assigned a severity rating ranging from 0 (“no usability problem”) to 4 (“usability catastrophe”).

### Competitive Analysis

A competitive analysis was conducted using a FigJam board (Figma, Inc) to generate strategic insights into potential alternative LMS platforms that could support the scalability and long-term delivery of the InPACT training program. As the program continues to expand, evaluating LMS options beyond its current Qualtrics-based delivery is essential for optimizing UX and infrastructure sustainability.

Three LMS platforms were selected for evaluation: Moodle (version 5.0), Teachable (version 3.3.0), and Thinkific (version 1.17). These platforms were selected based on their relevance to professional education delivery, scalability potential, and cost considerations. Each platform was evaluated through hands-on exploration from both the lecturer and learner perspectives. From the lecturer’s perspective, evaluators examined features related to course creation, content uploading, learner analytics, and collaboration among multiple contributors. From the learner’s perspective, the evaluation focused on progress tracking, feedback mechanisms, navigation, and alignment with usability heuristics such as visibility of system status (eg, progress bars and activity feedback) and user control and freedom (eg, back and exit buttons). Additional features relevant to both perspectives—including media integration, retention activities, and search or filtering capabilities—were also assessed.

The competitive analysis aimed to (1) identify LMS platforms that may better support InPACT’s future delivery needs and (2) inform potential enhancements to the current system by identifying effective design features used in other platforms. The analysis was conducted iteratively, recognizing the evolving nature of LMS technologies and prioritizing transferable insights over the selection of a single replacement platform.

### Data Analysis

Closed-ended survey items on the online survey (eg, yes or no responses and categorical frequency ratings) were summarized using descriptive statistics to characterize teacher demographics, module use, and perceived frequency of classroom activity implementation. Open-ended survey responses (eg, “What are some positives of the modules? Negatives?”, “If there is something you could change about the modules, what would it be?”, and “Is there anything else you’d like to add?”) generated free-text responses that were treated as qualitative data and analyzed using thematic analysis. All free-text responses were independently reviewed by members of the evaluation team to identify recurring patterns related to usability, content clarity, engagement, and technical functionality. An iterative process was used to group similar responses, refine theme definitions, and reach consensus on final themes. Representative quotes were selected to illustrate each theme and provide contextual depth to the findings. To protect participant confidentiality, pseudonyms were used for all quotes presented.

Data from the heuristic evaluation were analyzed by compiling all usability issues identified by individual evaluators and categorizing them according to the Jakob Nielsen 10 usability heuristics. Severity scores were determined through group discussion and consensus and were used to prioritize design recommendations.

Competitive analysis data were analyzed through a structured cross-platform comparison of LMS features relevant to usability and instructional design, including course creation, UX, and interactivity. Cross-platform patterns were synthesized to identify design features that could inform the optimization of the InPACT teacher training modules.

Overall, data analysis integrated descriptive quantitative summaries with qualitative thematic analysis and usability-focused evaluative techniques.

### Ethical Considerations

This study was approved by the institutional review board of the University of Michigan (HUM00192745). Prior to each interview, all participants provided verbal informed consent. Each participant was assigned a unique numerical identifier, and pseudonymized transcripts and personal data were stored separately on a secure Childhood Disparities Research Laboratory U-Drive, accessible only to authorized research team members. Participants received a US $25 gift card as compensation for their time.

## Results

### Structured Online Survey

A convenience sample of 11 schoolteachers (grade levels: kindergarten through 5th grade; teaching experience range: 3‐28 years; mean 18.0, SD 8.9) completed the structured online survey. Overall, teachers reported minimal technical or content-related barriers when completing the InPACT teacher training modules. Only 1 teacher reported experiencing technical issues, and no teachers reported confusion with the module content.

Use of supplemental materials varied across respondents. Approximately half of the teachers reported using a written program guide (PDF or physical version of the modules) alongside the online training, whereas the remaining teachers relied exclusively on the digital modules. Engagement with embedded supplementary links was mixed: 4 teachers reported frequent use, while others reported occasional use (n=5) or no use (n=2), often noting that the core module content was sufficient without additional resources.

Following the completion of the training, all teachers reported incorporating activity breaks into their classrooms, with most reporting doing so very often (n=8) and others somewhat often (n=3). Taken together, these findings indicate that the InPACT teacher training modules were clear, functional, and feasible to implement, with few reported barriers and flexible options for engaging with written materials and supplemental content.

Textbox 1 presents the themes derived from teachers’ open-ended survey responses, along with illustrative quotes drawn directly from participants’ free-text comments. Five major themes emerged from the thematic analysis of open-ended survey responses. The first theme concerned ease of use and accessibility. Most teachers reported that the modules were easy to navigate and understand, both in terms of the platform interface and the clarity of the content. The flexibility to complete the modules at one’s own pace was particularly appreciated, as many teachers noted they were unable to finish a module in a single sitting.

Textbox 1. **Themes emerging from thematic analysis of open-ended responses in the structured online survey. Pseudonyms are used for all quotes presented.**

**Theme 1: ease of use and accessibility**

- “I thought they were very easy and useful.” [P5, Janice Marquez]
- “Easy access.” [P7, Jose Jackson]
- “They were easy to follow and understand.” [P11, Lucy Johnson]

**Theme 2: content utility**

- “I’ve been using what I learned for years now.” [P12, Lauren Smith]
- “It had a lot of useful information.” [P3, Isabel Kim]
- “I have been using many activity breaks throughout my day and noticing significant improvement in student’s engagement, focus, but mostly happiness.” [P12, Lauren Smith]

**Theme 3: interactive and applied learning**

- “Examples that got us up and moving as we completed the modules.” [P4, Farrah Bandow]
- “More interactive activities/links.” [P3, Isabel Kim]

**Theme 4: engagement and presentation**

- “It was not presented in a very engaging way.” [P3, Isabel Kim]
- “I would be more concise and shorten them up.” [P13, Rhonda Barrales]
- “The video was hard to read.” [P2, Emily Canton]

**Theme 5: technical flexibility**

- “You had to wait before the module let you move forward.” [P11, Lucy Johnson]
- “The delay for the videos was sometimes longer than necessary so you had to wait before the module let you move forward.” [P10, Sarah Lee]

The second theme focused on the perceived utility of the content. Content utility is defined as how effectively content fulfills a user’s specific need, goal, or task, resulting in user satisfaction [41]. Participants described the modules as informative and relevant, highlighting their value as a resource for both learning new strategies and reinforcing prior knowledge. Specific content areas, such as classroom management techniques and safety considerations, were frequently cited as especially useful.

The third theme pertained to the interactive and applied nature of the learning experience. Teachers expressed appreciation for the modules’ interactive components and noted the practical application of the material in their classroom settings. They valued being able to convey information to students and implement the strategies directly in their teaching practice.

The fourth theme addressed engagement and presentation. While participants acknowledged the usefulness of the content, some indicated that the modules were overly dense and lacked engaging delivery. Suggestions for improvement included making the modules more concise and enhancing the presentation style to increase user engagement.

Finally, the fifth theme related to technical flexibility. A few participants reported technical issues, such as difficulties with video readability and excessive delays during video progression. To improve the overall UX, teachers recommended shortening the content and providing greater control over video playback, including options to skip or speed up sections as needed.

### Heuristic Evaluation

Table 2 presents the results of the heuristic evaluation conducted on the InPACT teacher training modules. A total of 14 usability issues across 9 of the Nielsen 10 heuristics were identified. For the heuristic of help and documentation, the training received a score of 2, indicating minor usability problems. For 6 heuristics—visibility of system status, match between the system and the real world, consistency and standards, recognition rather than recall, aesthetic and minimalist design, and help users recognize, diagnose, and recover from errors—the training received a score of 3, indicating major usability problems. Finally, the heuristics of user control and freedom and error prevention were rated with a severity score of 4, representing usability catastrophes. The heuristic of flexibility and efficiency of use—which emphasizes allowing both novice and expert users to interact effectively with a system, for example, by providing shortcuts or customizable features that make frequent actions faster—was not evaluated, as novice end users were not included in this evaluation.

**Table 2.** Heuristic evaluation conducted on Interrupting Prolonged Sitting with Activity (InPACT) modules.

Heuristic	Violation	Recommendation	Severity
1. Visibility of system status	The survey completion bar does not reflect the user’s progress, and there is no indication that progress is automatically saved.	Add a clear message that progress is autosaved and improve the survey bar accuracy.	3
2. Match between the system and the real world	The training modules can be seen to be more of a self-evaluation, rather than a training module, as there are “level of agreement” questions premodule and postmodule. Users would expect assessments, interactive elements, resources, and so on, when completing training modules.	Introduce interactive case studies that allow users to apply what they learned to a specific scenario. Add interactive assessments and resources for users to complete and/or learn more about as they complete the modules.	3
3. User control and freedom	Users cannot go back to previous screens without rewatching the entire video. No exit button for users to leave the module or return to a main menu (constraint with Qualtrics XM).	Allow users to navigate back without forcing them to rewatch completed videos. Add a clear exit or home button for better navigation.	4
4. Consistency and standards	Inconsistency in page titles (eg, “Do” and “Read” appear on some pages but not others).	Standardize headings across all modules for clarity.	3
5. Error prevention	No clear instruction that videos have a time Boolean, leading to confusion. Additionally, even if users fully watch the videos on a different video speed, they cannot advance to the next page.	Remove the time Boolean. Incorporate JavaScript to reflect the completion of videos. Inform users that they cannot go back without having to watch the video again.	4
6. Recognition rather than recall	Some pages contain large blocks of text without clear sectioning, making content hard to recall.	Break up text with headings, bullet points, and images.	3
7. Flexibility and efficiency of use	N/A^a^	N/A	N/A
8. Aesthetic and minimalist design	Some pages are very text-heavy, making content overwhelming. The final page is labeled as a “Summary of Responses” but does not clearly indicate next steps.	Incorporate images, videos, and better spacing to improve readability. Reword the title and include a clear call to action on what to do next.	3
9. Help users recognize, diagnose, and recover from errors	The form for entering an email address does not specify what a “valid email” format is. Users do not receive feedback if they have not completed a necessary step (eg, watching a video fully).	Provide clear instructions on acceptable email formats. Display an error message explaining why the next button is disabled.	3
10. Help and documentation	It is unclear if teachers need the program guidebook to complete the training. Check-in does not provide contact links for additional help.	Clearly state whether the book is required or optional. Include links to resources and a contact person for support.	2

a N/A: not applicable.

### Competitive Analysis

Table 3 highlights the strengths and limitations across the three evaluated LMS platforms. Moodle demonstrated strong capabilities for course creation and content organization, including support for multimedia integration, multiple courses per module, and clear navigation features, though it lacked consistent progress indicators, time estimates, and a dedicated “next” button. Teachable offered a responsive design across devices, collaborative course-building features, analytics, and interactive activities, but some aspects of content presentation were confusing, and students could bypass quizzes. Thinkific provided intuitive course creation, clear progress tracking, interactive quizzes with feedback, and helpful media labeling, but the course title occupied excessive screen space, limiting navigation efficiency. Collectively, these findings highlight the relative advantages and trade-offs of each platform, offering guidance for selecting or refining LMS solutions to optimize usability, learner engagement, and module scalability for the InPACT teacher training program.

**Table 3.** Competitive analysis, where course creation reflects how easily content can be developed and adapted; user experience captures usability, navigation, and accessibility; and interactivity reflects opportunities for engagement, practice, and feedback that support learning and implementation.

LMS^a^ name	Course creation	User experience	Interactivity
Moodle	(+^b^) Allows for the inclusion of slideshows, images, and videos within course content. (+) Ability to create multiple courses for each training module for less information overload.	(+) The user can filter and search through courses. (+) Tracking progress bar shows the user how far into the module they are. (+) There are organization and categorization of lessons (drop-downs for sublessons). (+) Indication of completed section (green dot). (+) There can be multiple pages within main sections to have less information presented all at once. (+) Clearly marked “back” and “exit” buttons, able to send the user back very easily and without confusion. (–^c^) Difficult to see progress on course, for some modules, no indications if a section is “completed” or not. (–) No time estimates. (–) No “next” button, user must navigate on their own with little to no information. (–) No progress on how many subsections are in each module, only using a percentage for progress.	(+) Moodle has learning retention activities, such as quizzes.
Teachable	(+) Similar process to Qualtrics XM, where the platform allows for blocks to add or remove items. (+) Ability to see users (students) and other analytics. (+) Collaborative features; multiple people can work on a Teachable course at once. (+) Option to bulk upload files (resources), videos, documents, and audio to create a lesson. (+) AI^d^ tools to include a section summary.	(+) Responsive design, so users can complete the modules on their laptop, tablet, and phones. (+) Students can see their progress and sections in the course. (–) Content section is a bit confusing with the circle icon.	(+) Interactive activities, such as quizzes and clicking on resources. (–) Students can complete a section without taking the quiz.
Thinkific	(+) Can easily select categories to create modules. (+) Ability to change settings on quiz. For example, requiring a passing grade or importing questions. (+) Easy to navigate course creation.	(+) Search bar within course allows easy navigation for the user. (+) Progress bar with numbers with the option to expand on sections. (+) Informs users about the type of media in each section (eg, video and quiz) and provides an estimated time to complete it. (+) Provides feedback on quiz when a user answers (correct or incorrect). (–) The title of the course takes up approximately 1/3 of the navigation section, and there is no way to minimize it.	(+) Interactive activities, such as quizzes and videos.

a LMS: learning management system.

b The plus sign (+) denotes observed strengths.

c The minus sign (–) denotes observed weaknesses.

d AI: artificial intelligence.

## Discussion

### Principal Results

Given the barriers teachers face in integrating physical activity into classroom routines—particularly related to confidence, stress, and limited time—the purpose of this study was to apply a HCD approach to evaluate and enhance the usability of the InPACT virtual teacher training modules. Using mixed methods—including a structured online survey, heuristic evaluation, and competitive analysis—we assessed teachers’ training experiences and identified key usability challenges and opportunities for improvement. Results from the structured survey revealed strong support for the modules’ content and structure, including perceived usefulness, flexibility, and applicability. However, teachers also noted that the modules could benefit from improved pacing, greater interactivity, and enhanced video controls. The heuristic evaluation uncovered 14 usability issues across 9 of the Nielsen 10 heuristics, including multiple major usability problems and a severity score of 4 (“usability catastrophe”) for user control and freedom. These findings pointed to critical design flaws that may hinder user engagement and reduce the likelihood of training completion. The competitive analysis compared three LMS platforms (Moodle, Teachable, and Thinkific) and highlighted key platform features—such as course creation flexibility, learner feedback mechanisms, and collaborative design tools—that could inform future iterations of the InPACT platform. Taken together, these results suggest that while the InPACT training modules are content-rich and well-aligned with teacher needs, several design and delivery improvements are needed to enhance usability, improve training uptake, and support the sustainable adoption of classroom physical activity practices.

Although this study focused primarily on individual-level factors related to teacher engagement with the training modules, the implementation of classroom-based physical activity interventions is influenced by multiple, interacting levels of context. At the inner setting level, factors such as administrative support, school culture, time constraints, and competing instructional priorities can directly shape teachers’ ability to apply training content in daily practice [23,42]. At the outer setting level, district policies, professional development requirements, and broader accountability pressures may further enable or constrain implementation [43,44]. Prior work in low-resource school settings has shown that even well-designed, evidence-based interventions may experience reduced fidelity or sustainment when organizational and system-level conditions are not aligned with implementation demands [44-46]. As demonstrated by Hasson et al [47], addressing these multilevel influences through leadership engagement, district partnerships, and adaptive implementation strategies is critical for translating individual-level training gains into sustained practice. Future efforts to scale teacher training modules should therefore incorporate complementary strategies that target school- and district-level barriers alongside individual professional development.

### Comparison With Prior Work

Prior research underscores the critical role of professional development in the successful implementation of school-based health programs [48]. Teachers are more likely to adopt and sustain new interventions when they receive training that is accessible, practical, and tailored to their classroom needs [49]. In the context of physical activity programming, effective professional development has been linked to improvements in teacher confidence, fidelity of program delivery, and student engagement [42,50,51]. Accordingly, the InPACT virtual teacher training modules were designed to serve as a central implementation strategy [52].

However, usability challenges identified in this evaluation suggest that the training modules fell short of best practices for online professional development. Using the Nielsen usability heuristic framework, the evaluation revealed 3 primary issues: lack of user control and freedom, inconsistency and lack of standards, and insufficient recognition rather than recall. Teachers described frustration with not being able to pause or skip segments of the training videos, which reflects poor user control and freedom. This limitation is especially problematic for time-constrained educators [47], who may prefer to pace their training across multiple sittings. Inconsistencies in video labeling and navigation—such as unclear module titles and nonuniform layouts—violated established design standards, making it harder for users to locate content, which can contribute to cognitive overload [53]. Finally, the training platform required users to remember previously viewed content with limited visual cues to support recall. This reflects insufficient recognition rather than recall, a heuristic that emphasizes reducing memory load by making options, actions, and information visible so that users can recognize what to do instead of relying on memory. Without visual scaffolding, such as clear navigation menus or progress indicators, the cognitive burden increases, and learnability suffers [54].

These usability shortcomings contrast with best practices from prior studies, which demonstrate that professional development resources should be easy to navigate, chunked into digestible segments, and provide clear cues to help users orient themselves within the training [40]. Addressing these issues is critical to improving the training experience for teachers and, in turn, increasing the implementation fidelity of classroom physical activity programming in schools.

### Recommendations for Enhancement of the InPACT Virtual Teacher Training Modules

A major usability barrier identified during the evaluation was teacher frustration with video replay requirements, particularly when navigating backward in a module. To address this issue, the evaluation team recommended removing the time-based Boolean constraint on embedded videos. Specifically, they proposed using JavaScript event listeners integrated with the YouTube application programming interface to track video play or pause actions and determine when a sufficient portion of the video has been viewed. This would allow users to resume or advance through content without having to rewatch full segments. In cases where this solution was not feasible within the current Qualtrics platform, the team recommended displaying clear notifications indicating that videos must be viewed in full before proceeding.

Additional enhancements were proposed to improve time management and overall UX. Teachers noted that the existing page-based progress bar was misleading, making it difficult to anticipate the time commitment. To improve clarity, the evaluation team proposed a content-based progress bar with labeled topic nodes, or alternatively, the inclusion of manual page indicators and estimated completion times.

To promote engagement and retention, the team suggested reducing dense text and incorporating visual aids and interactive components such as collapsible content sections, quizzes with feedback, real-world case studies, and downloadable action plans. These enhancements were aimed at strengthening active learning and implementation fidelity. Navigation improvements were also prioritized. Teachers expressed difficulty tracking progress across modules, so a standardized end-of-module message with clear next steps (eg, “To continue to the next module, click here”) was recommended. Inconsistencies in how modules referenced the companion program guide led to confusion, prompting a recommendation to standardize these references and clearly indicate when guide components are optional.

To implement these enhancements and support scalability, we partnered with Michigan Virtual, a statewide leader in accessible professional learning [54]. Michigan Virtual committed to redesigning and developing the revised modules using their Brightspace LMS, ensuring compliance with Web Content Accessibility Guidelines and Americans with Disabilities Act standards. Their instructional designers identified necessary content updates, helped revise training materials, and provided technical support. Michigan Virtual also offered to host the updated modules on its Professional Learning Portal, manage State Continuing Education Clock Hours processing, and share educator completion data with the InPACT team. This partnership is expected to ensure a high-quality, accessible, and scalable professional development experience for educators statewide.

Preliminary data from the revised InPACT teacher training modules hosted on Michigan Virtual indicate strong early uptake, engagement, and performance during the first eight months following release. As of April 7, 2026, 2061 educators have enrolled in the training with 1138 participants earning certificates of completion, reflecting substantially greater reach and completion volume than the prior Qualtrics-hosted training (35 teachers over a 3-year period). Knowledge acquisition was assessed using standardized postmodule quizzes embedded within each training module. Across these module quizzes, participants demonstrated high mastery of content, with an average score of 90.6% (SD 2.7%), and required relatively few attempts to achieve passing scores (mean 1.5, SD 0.2 attempts per module). Collectively, these preliminary findings suggest that the revised training—coupled with a more accessible delivery platform—has markedly improved scalability, engagement, and learning outcomes.

Partnerships played a central role in guiding the enhancements to the InPACT teacher training modules. Engaging a diverse set of collaborators—including classroom teachers, instructional designers, accessibility specialists, and statewide professional learning organizations—was essential for identifying usability challenges, prioritizing improvements, and ensuring that module revisions were both practical and scalable. These partnerships enabled the co-development of solutions that enhanced navigation, interactivity, and accessibility, while ensuring alignment with accessibility and disability standards. For teams with limited budgets, leveraging existing relationships, focusing on high-impact usability improvements, and incorporating low-cost accessibility strategies—such as consistent page structure, clear labeling, and visual scaffolding—can help maximize impact. Additionally, integrating clear data visualization and feedback mechanisms, alongside ongoing communication and iterative feedback with end users, is recommended to promote engagement, learning retention, and sustainable implementation of virtual professional development programs.

### Strengths and Limitations

This study has several notable strengths. First, we used a mixed methods usability evaluation that incorporated both end-user feedback and expert recommendations to guide improvements to the InPACT teacher training modules. Second, the evaluation was conducted by a multidisciplinary team with expertise in instructional design, educational technology, and human-computer interaction. Finally, our collaborative and HCD approach fostered partnerships between teachers, evaluators, and implementation support organizations, enabling the co-development of a more accessible, engaging, and scalable professional learning experience for educators across Michigan.

Limitations of this study also warrant consideration. First, teacher feedback was based on self-report and retrospective recall, which may be subject to recall bias or social desirability bias and may not fully capture real-time usability challenges encountered during module completion. Although self-report data are commonly used in early-stage usability and implementation research to efficiently gather end-user perspectives, these methods may overlook nuanced interaction patterns, moment-to-moment frustration points, or navigation errors that occur during actual use [55]. Future usability evaluations that integrate observational and system-level data with teacher self-reports will be critical for identifying real-time barriers and optimizing training engagement and completion.

Second, the participant sample consisted primarily of teachers who were already implementing the InPACT program. While this was appropriate for the study’s aim of evaluating the usability of existing training modules, these participants may have had greater familiarity with the program content and underlying concepts than educators new to InPACT. Future evaluations should include educators unfamiliar with InPACT to examine initial usability, onboarding experiences, and barriers faced by first-time users.

Third, this study did not assess whether the usability improvements applied in the revised modules translated into changes in teacher self-efficacy, adoption of classroom activities, or fidelity of implementation. Future research is needed to determine whether usability-informed redesigns of the InPACT teacher training modules lead to measurable improvements in teacher self-efficacy, training completion, adoption, and implementation fidelity.

### Conclusions

Most teachers in the United States do not implement classroom physical activity breaks with the frequency or fidelity recommended to improve student health and academic outcomes [14,15]. Virtual training modules offer a scalable approach to build teacher capacity, but their effectiveness depends not only on high-quality content but also on user-centered design and usability. In this study, a combination of heuristic evaluation, competitive analysis, and end-user surveys was used to identify and address barriers to teacher engagement with the InPACT virtual teacher training modules. Key recommendations focused on improving navigation, video accessibility, and visual appeal to reduce user frustration and enhance the learning experience. Applying an HCD approach enabled collaboration between evaluators, developers, and end users to optimize virtual training for broader adoption. Future work should examine whether these HCD-informed revisions lead to measurable improvements in teacher self-efficacy, training completion, classroom physical activity program adoption, implementation fidelity, and student outcomes.

## Supplementary material

10.2196/83427 Multimedia Appendix 1 Structured online survey instrument.

## Abbreviations

HCD: human-centered design
InPACT: Interrupting Prolonged Sitting with Activity
LMS: learning management system
UX: user experience
