# Supplementary material for: Improving Usability of the Interrupting Prolonged Sitting With Activity Virtual Teacher Training Modules: Case Study
Source: JMIR Hum Factors. 2026 Apr 21;13:e83427. doi: 10.2196/83427 (PMC13099026; doi:10.2196/83427)
Supplement: Multimedia Appendix 1 [file humanfactors-v13-e83427-s001.docx]

The following questions were administered to elementary school teachers who completed at least one virtual InPACT Teacher Training Module. All participants received the same survey

**Demographic Information**

1. What grade do you teach?
2. How long have you been teaching?

**Module Experience and Usability**
3. Did you run into any issues with the modules?
4. Were any of the modules confusing?

- Yes
- No

1. What are some positives of the modules? What are some negatives? (Open-ended)
2. Did you use the physical Program Guide or the PDF version while completing the online modules?
3. Did you often use the links on the training pages that led to more information?
   - Yes
   - No
   - Sometimes
4. If there is something you could change about the modules, what would it be? (Open-ended)

**Implementation**
9. After completing the modules, how often do you use ACTivity in your classroom?

- Very often
- Somewhat often
- Rarely

**Additional Feedback**
10. Is there anything else you’d like to add? Thank you for your response! (Open-ended)
